# *A new resorbable device for ligation of blood vessels - A pilot study*

**DOI:** 10.1186/1751-0147-53-47

**Published:** 2011-07-08

**Authors:** Odd V Höglund, Ragnvi Hagman, Kerstin Olsson, Jonas Mindemark, Niklas Borg, Anne-Sofie Lagerstedt

**Affiliations:** 1 Department of Clinical Sciences, Swedish University of Agricultural Sciences, Box 7054, SE-750 07 Uppsala, Sweden; 2 Department of Anatomy, Physiology and Biochemistry, Swedish University of Agricultural Sciences, Box 7011, SE-750 07 Uppsala, Sweden; 3 Department of Materials Chemistry, Uppsala University, Box 538, SE-751 21 Uppsala, Sweden; 4 Radi Medical Systems, Palmbladsgatan 10, SE-754 50 Uppsala, Sweden

## Abstract

**Background:**

During surgery, controlled haemostasis to prevent blood loss is vital for a successful outcome. It can be difficult to ligate vessels located deep in the abdomen. A device that is easy to use and enables secure ligatures could be beneficial. Cable ties made of nylon have been used for ligation but the non-resorbable material caused tissue reactions. The objective of this study was to use a resorbable material to construct a device with a self-locking mechanism and to test its mechanical strength and ligation efficiency.

**Methods:**

The device was manufactured by injection moulding of polydioxanone, a resorbable polymer used for suture materials. Polydioxanone with inherent viscosities of 1.9 dL/g and 1.3 dL/g were tested. The device consisted of a perforated flexible band which could be pulled through a case with a locking mechanism. After a first version of the device had been tested, some improvements were made. The locking case was downsized, corners were rounded off, the band was made thicker and the mould was redesigned to produce longer devices. Tensile tests were performed with the second version.

The first version of the device was used to ligate the ovarian pedicle in a euthanized dog and to test echogenicity of the device with ultrasound. Compression of vessels of the ovarian pedicle was examined by histology. Both versions of the device were tested for haemostasis of and tissue grip on renal arteries in six anaesthetised pigs.

**Results:**

The tensile strength of the flexible band of the devices with inherent viscosity of 1.9 dL/g was 50.1 ± 5.5 N (range 35.2-62.9 N, *n *= 11) and the devices with inherent viscosity of 1.3 dL/g had a tensile strength of 39.8 ± 8.1 N (range 18.6-54.2 N, *n *= 11). Injection moulding of the polymer with lower inherent viscosity resulted in a longer flow distance.

Both versions of the device had an effective tissue grip and complete haemostasis of renal arteries was verified. The device attached to the ovarian pedicle could be seen with ultrasound, and vessel compression and occlusion were verified by histology.

**Conclusions:**

Tests of functionality of the device showed complete haemostasis and good tissue grip. Devices with a band of sufficient length were easily applied and tightened in tissue.

## Background

Bleeding associated with surgery is a potentially serious complication. Haemostasis to prevent blood loss is commonly achieved by tying a surgical thread around the blood vessel. Vessels localized deep in the abdomen can be difficult to ligate [1]. A device that enables secure ligatures that are easily applied could be beneficial.

The use of non-resorbable nylon cable ties for haemostasis in soft tissue surgery has been described [2-8]. However, non-resorbable materials used for ligation purposes and left permanently in tissue, can cause complications such as fistulas and granulomas [9-11]. The advantages of cable ties for ligation purposes were ease of application, quick ligation procedures and good "knot" security [6-8]. If a device, based on the construction of a self-locking loop, could be made of a resorbable material it could minimize the risk of adverse tissue reactions but potentially maintain the advantages experienced with traditional cable ties.

The aim of this study was to develop a device of a resorbable material, which enables reliable and easy ligation of blood vessels.

## Methods

### Design of the device

**Figure 1:**
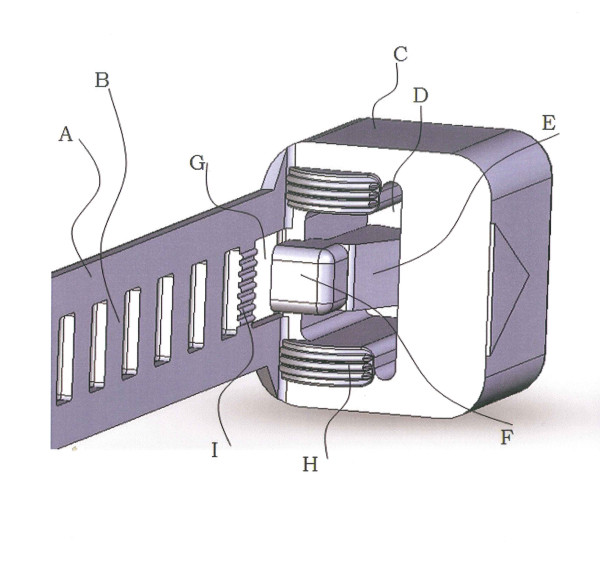
**Design of device**. The resorbable device comprised an elongated, flexible band (A) in part perforated (B) that formed a ladder structure and was connected to a locking case (C) that had a channel (D) dimensioned for reception of the band (A). A locking device (E) was configured to interlock the perforated band (B) when the band (A) was pulled through the channel (D) and a reverse-motion brake was formed. A protrusion (F) and a matching receiver (G) were arranged at the channel entrance and in the band-locking case interface. The receiver (G) was dimensioned for inclusion of the protrusion (F) when the band (A) was close to fully pushed into the locking case (C). Tissue-engaging ridges (H) were arranged on the side of the locking case of the device and on the front side (I) of the band.

To construct the device computer-aided design (Solidworks, Dassault Systèmes SolidWorks Corporation, Concord, USA) was used. The dimensions were selected according to the desired strength, possible flow distance during injection moulding and ability of the device to fully close. The device consisted of a flexible band, in part perforated, and a case with a locking mechanism where the band could be introduced and pulled through. Details for tissue engaging properties were added to the locking case and the band surface (Figure 1). A protrusion at the connection between locking case and band formed a cog merging with the first hole in the band when the band was fully pulled through. The tissue-engaging ridges at the side of the locking case aligned towards the band surface and connected with tissue caught in the loop.

### Design of moulds for injection moulding

**Figure 2:**
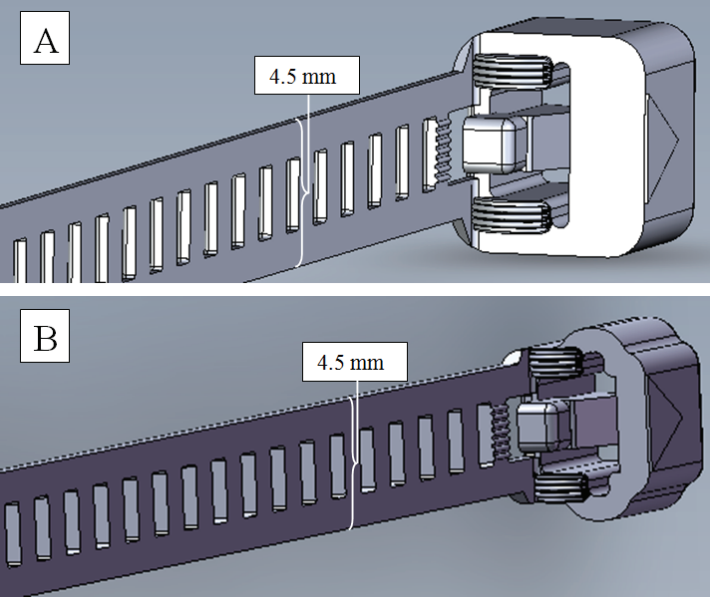
**The two versions of the resorbable device used in the study**. Figure 2A. The first version of the device had a larger locking case and shorter band. Polydioxanone with inherent viscosity of 1.9 dL/g was used. Figure 2 B. The second version of the device had a smaller locking case and longer band. Polydioxanone with inherent viscosity of 1.3 dL/g resulted in a longer flow distance.

The device was manufactured in purpose-built steel moulds (Mecdon, Laxå, Sweden). During development a redesigned second version's mould was made to improve the device. The second version of device had a downsized locking case, thinner walls and rounded-off corners. Consequently it contained less material than the first version (Figure 2). The inlet for the polymer into the second version's mould was adjusted and the thickness of the flexible band increased from 0.55 mm to 0.65 mm which created bands of greater length and strength. Controlled heating was used in the second version's mould for improvement of flow distance during injection moulding.

### Injection moulding of polydioxanone

Polydioxanone (Resomer^® ^X, Boehringer Ingelheim Pharma GmbH, D-55216 Ingelheim, Germany) was chosen for the material of the device, a synthetic resorbable polymer which is widely used for suture materials and implants since many years. It is flexible and elastic and its induction of inflammatory reactions is minimal. It is degraded through hydrolysis and may be completely resorbed within 6-12 months [12,13].

The polymer was heated above melting temperature and injected into the mould using an in-house built injection moulding machine. High pressure was maintained after injection and crystallization was allowed to occur for about one minute before the mould was opened. Polydioxanone of inherent viscosity of 1.9 dL/g was used for injection moulding in the first version's mould. Two batches of polydioxanone with inherent viscosities of 1.9 and 1.3 dL/g respectively were tested for injection moulding in the second version's mould.

### Tensile testing

The tensile strength of the flexible band was measured using a 5544 Single Column Testing System (Instron, USA). Samples were prepared by cutting away the locking case of the device and the solid part of the flexible band, leaving approximately 4 cm of the perforated band. The samples were clamped 1 cm from each end and pulled to break at a rate of deformation of 40 mm/min. The tensile strength was determined as the maximum failure load. Eleven samples manufactured from each of the polymer batches of polydioxanone were tested. All tested devices were of the second version. The level of statistical significance was defined as *p *< 0.05 (Student's t-test, equal variances not assumed).

### Animals

One dog weighing 20 kg, which was euthanized prior to the test due to reasons not associated with this study, and six pigs weighing 25-28 kg, which were anaesthetised for other reasons at Uppsala University Hospital, Sweden, were used in the study. The Uppsala Animal Ethics Committee, Sweden, approved this additional test (permission number C172/8).

### Test of the devices in tissue

#### Ovarian pedicle, imaging and histology

An incision was made from approximately 2 cm cranial of the umbilicus along the midline of the abdomen in the dog. The *linea alba *was incised, the abdomen opened and the uterine horns and ovaries were localized. A hole was made in the broad ligament close to the left ovary. A loop was formed around the ovarian pedicle and the band was pushed into the locking mechanism (first version of the device). The loop was tightened, compressing the tissue inside the loop. The surplus band extending beyond the channel exit in the locking case was cut off.

The echogenicity of the device was examined with ultrasound (Sequoia 512, Acuson, Siemens AG Medical Solutions, Germany) with an 8.0 MHz linear probe. The probe was placed on the abdomen after application of ultrasound gel on the skin. After the investigation the device with adjacent tissue was removed and embedded in resin (Historesin, Leica Microsystems Nussloch GmbH, Germany). After dehydration in increasing concentrations of ethanol, followed by increasing concentrations of water-soluble resin, the sample was embedded in 100% resin. Sections were cut using a microtome (Leica RM 2165, Leica Instruments GmbH, Germany) with glass knives as close as possible to the device. The samples were mounted on glass and stained with hematoxylin and eosin (HE).

#### Test of haemostasis and tissue grip of renal arteries in six pigs

The abdomen was opened midway along the *linea alba *and both renal arteries were free-dissected in two (first version) and four pigs (second version), respectively. A loop was formed around the artery with the device and the loop was tightened causing compression of the vessel. When the band was fully pulled through the locking case of the device, the artery was cut between the kidney and the device about 0.5 cm from the device. The renal artery with the attached device was inspected for haemostasis for about 5 minutes. The ability of the device to withstand a ligature slip-off was tested by applying a force of 10 N using a dynamometer attached to the device.

Arterial blood pressure was registered by invasive continuous measurements in the femoral artery of the pigs and was recorded once a minute (SC 9000XL, Siemens Medical Solutions, USA). The least square means of systolic and diastolic blood pressures during ligation were calculated.

## Results

### Injection moulding of polydioxanone

The mould for version one resulted in short devices whereas the modified design of mould for version two improved manufacturing results. The polymer with an inherent viscosity of 1.9 dL/g resulted in a shorter flow distance in the moulds and made the flexible band too short for optimal handling during surgery. The polymer with an inherent viscosity of 1.3 dL/g resulted in a longer flow distance, 10 cm, and the second version's mould was filled. A higher temperature of the melted polymer and mould facilitated flow distance in the mould but a higher temperature of the mould increased the time needed for crystallization. At least one minute was necessary for crystallisation of the polymer to occur. The material of the device expanded if sufficient crystallization had not occurred when the mould was opened, which caused constriction of the channel through the locking case.

### Tensile testing

**Figure 3:**
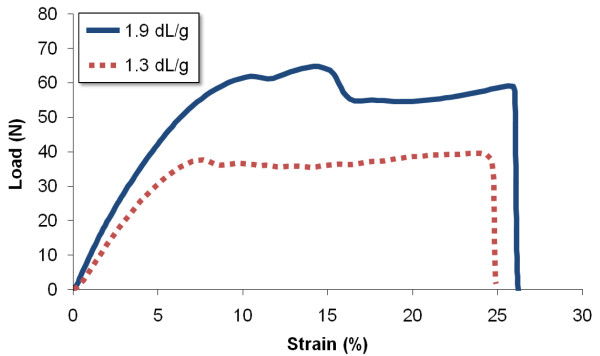
**Tensile testing**. Typical load versus strain curves for the devices at a rate of deformation of 40 mm/min.

The tensile strength of the device was assessed by measurement of the maximum failure load. The mean tensile strength (mean ± 95% confidence limits) for devices manufactured from the 1.9 dL/g batch was 50.1 ± 5.5 N (sample range 35.2-62.9 N). For devices manufactured from the 1.3 dL/g batch, the mean tensile strength was 39.8 ± 9.1 N (sample range 18.6-54.2 N). The difference between these two measurement series was statistically significant (*p *= 0.031). Typical mechanical responses to this test can be seen in Figure 3.

### Tests of first and second version of the device in tissue

**Figure 4:**
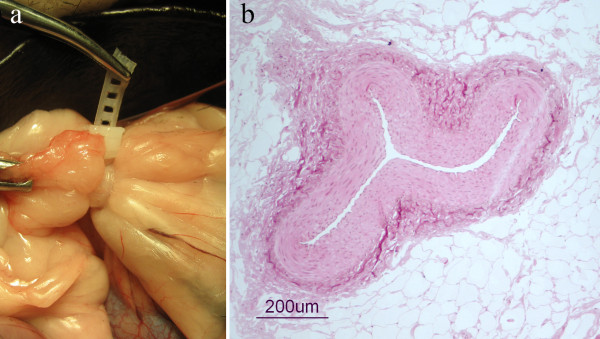
**Tissue test**. 4a. Ligation of the ovarian pedicle in a dog cadaver using the first version of the device. 4b. Histologic image of the compressed artery in the ovarian pedicle adjacent to the device.

The flexible band of the first version of device (1.9 dL/g) was too short for easy application around the tissue and therefore extended with a thread whereas the second version of the device, made from the lower inherent viscosity material (1.3 dL/g), was sufficiently long. The loop of the device, however, was easily tightened with one hand. When the ovarian pedicle was ligated one hand was used to hold the ovary and secure a proper distance between the loop of the device and the ovary. The other hand tightened the loop of the device and the tissue inside the loop was compressed (Figure 4). Ultrasonographic examination after application of the device in the dog showed that the device was hyperechoic and caused an obvious acoustic shadowing. The histological examination of the compressed tissue of the ovarian pedicle showed occlusion of vessels (Figure 4).

**Figure 5:**
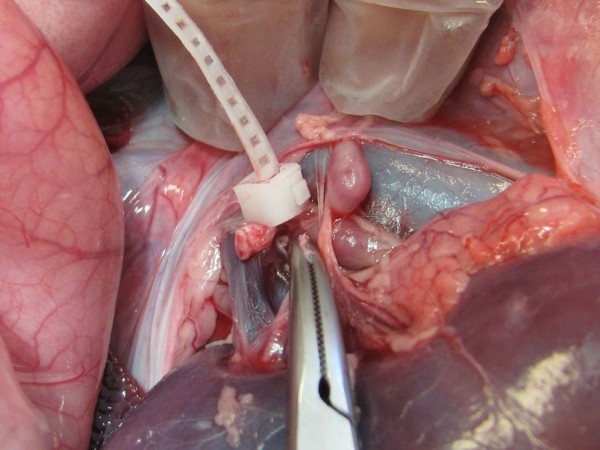
**Haemostasis and tissue grip of renal artery**. The renal artery of an anaesthetised pig ligated with the first version of the device.

Complete haemostasis of all twelve arteries was obtained (Figure 5) and verified for five minutes. The devices were securely locked into the tissue, *i.e*. no sliding along the vessels was observed. The systolic and diastolic blood pressures during ligation were 104/64 mmHg.

## Discussion

The production of the resorbable device was successful and the tests of functionality in animal tissues showed that an effective tissue grip and complete haemostasis were achieved.

The use of polydioxanone of higher inherent viscosity (1.9 dL/g) increased the tensile strength of the device but was a limitation in the manufacturing process due to shorter flow distance. The flow distance can be increased with higher temperature of the mould, but then the time needed for crystallization may have to be shortened by cooling the mould. The optimal process temperature during injection moulding is dependent of desired flow distance, diameter of the mould's channels, inherent viscosity of the polymer, its melting point and injection pressure. A higher temperature during the manufacturing process and lower inherent viscosity (1.3 dL/g) facilitates flow but temperature has to be balanced against detrimental change of properties of the polymer [14]. The inherent viscosity of the polymer also affected mechanical characteristics of the device, as would be expected by the difference in molecular weight. The results of the tensile test are similar to other studies of suture material (polydioxanone 3-0) at time zero [15,16] but comparisons should be done cautiously as the methods used are not identical.

Both versions of the device were tested on free-dissected renal arteries. Ligatures on the renal artery in connection with nephrectomy must be strong enough to prevent bleeding due to the high blood pressure in these vessels. Two ligatures [17] or two clips on the renal artery [18,19] are therefore regularly applied for security reasons. The device, however, has a built-in double security and yields two ligations similar to a double ligature. The renal arteries in the pigs, pressed towards the protrusion and the ridges, were squeezed into a zigzag pattern which created haemostasis. A double ligation is achieved by pressing the artery against the two ridges, one on each side of the locking case. If one side of the band breaks the flexible band cannot be reversed through the locking case. The construction also prevents the device to slide off the tissue. The renal arteries of the pigs were only observed for a short time, but complete haemostasis was obtained. Sufficient length of the device is important for ease of application in tissue and longer devices were successfully manufactured with the mould of the second version of the device and a lower inherent viscosity. A smooth and easy passage of the band through the locking case with a trustworthy mechanical performance of the locking mechanism of the device is essential for safety.

In the dog, the device effectively compressed the vessels together with the surrounding fatty tissue in the ovarian pedicle as verified by histology. The size of the self-locking loop of the device suggests that it may be used for ovarian pedicles regardless of breed size. The device could be seen on ultrasound imaging. The polymer may in the future be blended with a radiopaque substance to facilitate x-ray imaging [20]. There were some study limitations. The degradation effect on the polymer during the manufacturing process was not investigated and the tensile test was restricted to the perforated flexible band of a limited number of devices. As tests under hydrolytic conditions over time were not performed, conclusions are limited to initial strength.

## Conclusions

A novel injection moulded device made of the resorbable polymer polydioxanone was constructed. Tests of functionality of the device showed complete haemostasis and good tissue grip. Devices with a band of sufficient length were easily applied and tightened in tissue.

## Competing interests

The first author is owner of granted patent of the design of the device. The other authors declare that they have no competing interests.

## Authors' contributions

OVH conceived the idea of the device and its design, participated in injection moulding, the design of the study and drafted the manuscript. JM performed tensile tests, statistical analysis and helped to draft the manuscript. NB advised and performed injection moulding. RH, KO and ASL participated in the design of the study, its coordination and helped to draft the manuscript. All authors read and approved the final manuscript.
